# Use of Oral Bisphosphonates in Primary Prevention of Fractures in Postmenopausal Women: A Population-Based Cohort Study

**DOI:** 10.1371/journal.pone.0118178

**Published:** 2015-04-10

**Authors:** Jordi Real, Gisela Galindo, Leonardo Galván, María Antonia Lafarga, María Dolors Rodrigo, Marta Ortega

**Affiliations:** 1 Institut Universitari d’Investigació en Atenció Primària Jordi Gol (IDIAP Jordi Gol), Lleida, Spain; 2 School of Medicine and Health Sciences, Universitat Internacional de Catalunya, Sant Cugat del Valles, Spain; 3 Primer de Maig Center, Institut Català de la Salut, Lleida, Spain; 4 Catalan Health Departament, Lleida, Spain; 5 Bordeta-Magraners Center, Institut Català de la Salut, Lleida, Spain; 6 Cappont Center, Institut Català de la Salut, Lleida, Spain; Universidad de Valladolid, SPAIN

## Abstract

Oral bisphosphonates are first-line drugs in the treatment of osteoporosis under most guidelines, and have been shown to decrease risk of first fracture only in asymptomatic vertebral fractures and in clinical trial populations that are generally very different from the general population.

**Objective:**

To compare incidence of first osteoporotic fracture in two cohorts of postmenopausal women, one treated with bisphosphonates and the other only with calcium and vitamin D.

**Design:**

Retrospective population cohort study with paired matching based on data from electronic health records.

**Setting:**

Women aged 60 years and older in 2005, from 21 primary care centers in a healthcare region of Spain.

**Participants:**

Two groups of women aged 60 years and older (n = 1208), prescribed either calcium and vitamin D (CalVitD) or bisphosphonates (BIPHOS) with or without calcium and vitamin D, were compared for the end point of first recorded osteoporotic-related fracture, with 5-years follow-up.

**Main Outcome Measure:**

Incidence of first fracture: Vertebral fracture, osteoporosis with pathological fracture, fracture of the upper humeral epiphysis, fracture of the lower radial epiphysis, or femur fracture.

**Results:**

Estimated 10-year risk of fracture was 11.4% (95% confidence interval: 9.6 to 13.2), 11.8% (9.2 to 14.3) in the BIPHOS group and 11.1% (8.6 to 13.6) in the CalVitD group. No significant differences were found between groups in total fractures (Hazard ratio = 0.934 (0.67 to 1.31)) or location (vertebral, femoral, radial or humeral).

**Conclusions:**

In postmenopausal women, bisphosphonates have not been shown to better decrease risk of first fracture compared with calcium and vitamin D therapy alone.

## Introduction

Osteoporosis is clinically characterized by a loss of bone mass and changes in bone structure that cause fragility and contribute to the appearance of fractures, mainly of the vertebrae, femoral neck, and wrist [1]. The condition began to be defined in the 1990s, coinciding with the development of densitometry, and since then has been classified as a disease [2].

In 1994, a World Health Organization report classified women as healthy or diseased according to their bone mineral density (BMD) value, comparing them with an average 30-year-old woman [3]. This led to classify many healthy women as having osteoporosis and starting drug therapies in women who were not at risk of future fractures.[4] At present, a decline in BMD is considered a risk factor, not an indication of the disease, and patients whose only symptom is low BMD, determined by computed tomography (CT) scan, are not labelled as having osteoporosis [2].

In clinical practice, it is important to identify patients with a high risk of fracture and decide who should be treated and how [5,6]. In daily practice, however, decision-making is difficult because of many uncertainties, heterogeneity in clinical guidelines published by the various scientific societies [7], and even differences among doctors in the same country and medical specialty [8]. To decrease this variability, tools have been introduced to estimate the risk of future fractures, taking into account the various risk factors; the two main scales are FRAX [9], and QFRACTURE [10,11]. Both scales incorporate history of fracture, family history of hip fracture, underweight (BMI<18.5 kg/m2), smoking, alcohol consumption, and glucocorticoid treatment.

Of the available treatment options, bisphosphonates have the longest track record, have been the most studied, and are the least expensive drug choice. Meta-analysis of the different bisphosphonates has repeatedly shown a decline in new fractures among postmenopausal women in secondary prevention, defined as women with previous fracture and women without fractures and at least 2 SD values below the peak bone mass or older than 62 years when these data were not available. However, no treatment benefit has been observed in primary prevention except in the case of asymptomatic morphometric spinal fractures in women taking alendronate [12–14].

In the general population and in our setting, few studies have analysed the impact of osteoporosis treatments. One of these, an ecological study in Galicia by Guerra-García, observed that the number of units of anti-resorptive agents dispensed by pharmacies nearly doubled from 2004 to 2008 but there was no decline in the number of femoral fractures, which are the most serious osteoporotic fractures and have the worst consequences for patients [15]. Another ecological study using data from all the Spanish public health system detected a slight decrease between 2002–2008 years of adjusted hip fracture rates in women over 50 years (from 4.1 to 3.91 per 1000) in contrast with the sharp increase in the bisphosphonates consumption, multiplied by 5, in the same time period [16]. The 10-year cost of avoiding one hip fracture ranges from 54,134 to 84,287 euros with alendronate and 67,853 to 173,748 euros with risedronate treatment [17].

In daily clinical practice, anti-resorptive therapy is often prescribed as primary prevention in women younger than 60 years. Sanfélix-Gimeno commented on “the peculiar panorama” of osteoporosis management in our country, where excessive diagnostic testing is ordered and the treatment pattern is to prescribe anti-resorptive drugs and calcium plus vitamin D supplements more frequently for younger women with low risk than for older patients with high risk [18].

In 2009, a meta-analysis of oral bisphosphonates use in women older than 65 years showed a 24% reduction in osteoporotic fracture risk, a lower benefit than has been indicated in some clinical trials and highly associated with treatment adherence [19]. Another study identified an increased risk of atypical femoral fractures with this therapy [20]. Nonetheless, the results of these studies cannot be generalized to the population treated in usual clinical practice. It has been estimated that only 20% of the participants in randomized clinical trials are representative of the general population with osteoporosis [21].

Although oral bisphosphonates are first-line drugs for osteoporosis treatment under most guidelines [22,23], it is not clear that the associated reduction in the risk of first osteoporotic fracture is cost-effective in the general population. Maintaining long-term anti-resorptive therapy when its effectiveness is doubtful is a waste of resources. In addition, one should be very cautious in primary prevention because interventions have secondary effects; these must have highly conclusive evidence of effectiveness and long-term safety because they target large segments of the population and healthy individuals [24].

Despite the high social and healthcare impact of osteoporosis, the efficiency of the drugs most commonly prescribed in Spain for the prevention of osteoporotic fractures has not been sufficiently evaluated [25]. The aim of the present study was to estimate the incidence of first osteoporotic fracture in a cohort of postmenopausal women treated with bisphosphonates, compared with women treated only with calcium and vitamin D, using a population database of retrospective clinical records and 5-year follow-up.

## Material and Methods

### Study population

The study was carried out in a cohort of women aged 60 years and older assigned to any of the 21 healthcare centres in the Health Region of Lleida (HRL) belonging to the Spanish National Health Service, with universal coverage during the studied period. In 2005, the HRL covered a total population of 360,489, of which 42,234 were women aged 60 years and older.

### Design

**Fig 1:**
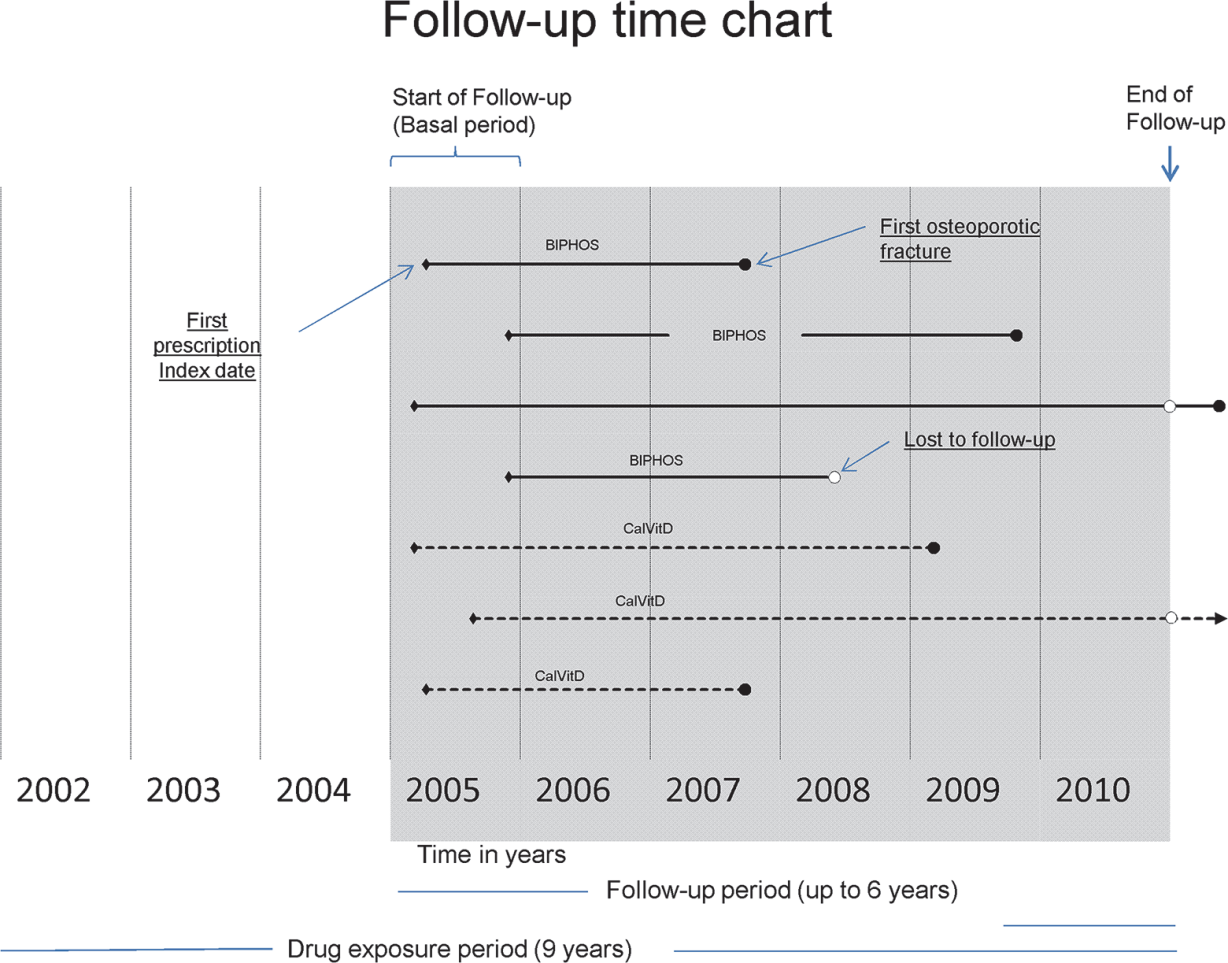
Follow-up time chart.

We designed a retrospective population cohort study with 5-year follow-up, matching two cohorts by clinical characteristics and drugs taken, based on the HRL database of electronic health records. The research potential these data provide for population studies has been previously described [26]. All patients registered in the HRL database who were at least 60 years old at the time of inclusion and taking calcium, vitamin D, and/or bisphosphonates under their doctor’s prescription were included in the study. The date of treatment initiation was considered the date that a pharmacy dispensed the first prescription (index date), according to the official pharmaceutical database (Fig. 1).

**Fig 2:**
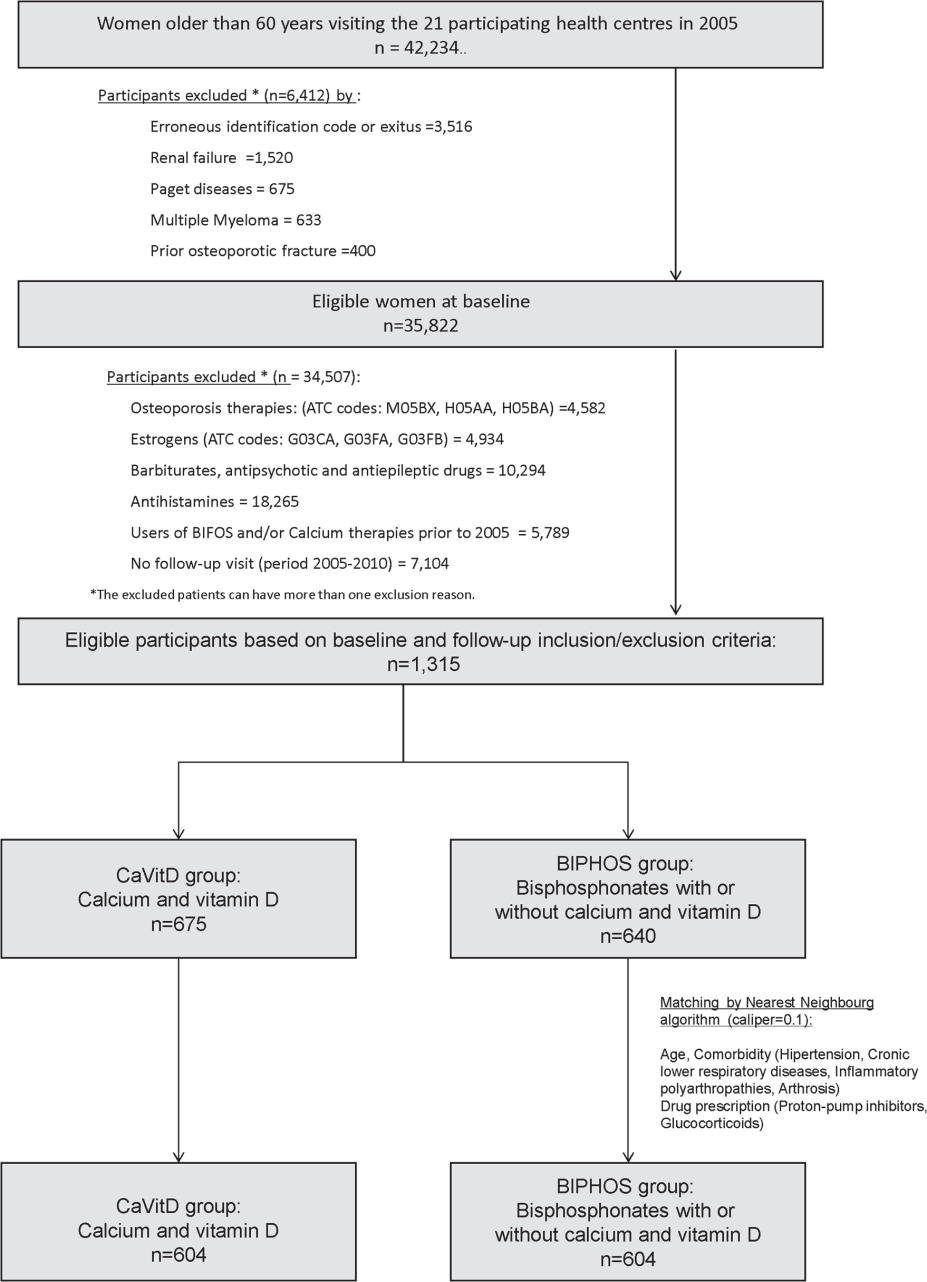
Flow chart showing the participants’ selection process.

Exclusion criteria (Table 1 and Fig. 2) included previous treatment (before index date at 2005) with drugs that modify bone metabolism (bisphosphonates and/or calcium, vitamin D, oestrogens, calcitonin, parathyroid hormone, strontium ranelate, or raloxifene); known history of osteoporotic fracture, kidney failure, Paget disease, or multiple myeloma; enrolment in the HRL database after 2002; and lack of contact with their HRL doctor during the follow-up period (2005–2010).

**Table 1:** Study variables.

Variable type		Codes: Source	
	Definition (Time)	ICD-10: Primary care	ICD-9: Hospital
**Primary outcome (between 2005 and 2010)**			
	Vertebral fracture: fatigue / unspecified	M48.4 / T08	733.13/ 805.xx
	Osteoporosis with pathological fracture	M80	733.11–733.19
	Fracture of the upper humeral epiphysis	S42.2	812.xx/ 733.11
	Fracture of the lower radial epiphysis	S52.5	813.42/ 813.52/733.12
	Fracture of the lower radial epiphysis	S52.5	813.42/ 813.52/733.12
	Femur fracture	S72	820.xx-821.xx/733.14/733.15
**Comorbidity, basal at index date**			
	Diabetes mellitus	E10-E14	
	Behavioural syndromes associated with physiological disturbances and physical factors	F50-F59	
	Extrapyramidal and movement disorders	G20-G26	
	Episodic and paroxysmal disorders	G40-G47	
	Hypertensive diseases	I10-I15	
	Ischemic heart diseases	I20-I25	
	Cerebrovascular diseases	I60-I69	
	Chronic lower respiratory diseases	J40-J47	
	Inflammatory polyarthropathies	M05-M14	
	Arthrosis	M15-M19	
**Exclusion diseases (at index date)**			
	Renal failure	N17-N19	
	Paget's diagnoses	M88	
	Multiple myeloma	C90.0	
**Drugs**		**ATC: Pharmacy**	
	**Primary**		
	Bisphosphonates, with/without Calcium	M05BA, M05BB	
	Calcium + vitamin D	A11CC A12AA A12AX	
	**Secondary drugs**		
	Antidepressants	N06AA, N06AB,N06AG,N06AX	
	Proton-pump inhibitors	A02BC	
	Glucocorticoids	H02AB	
	Benzodiazepine	N05AH, N05AL, N05BA, N05CD NC5CD, N05CF	
	Opiates	N02AA,N02AB,N02AC,N02AE,	
	**Exclusion (prior index date)**		
	Osteoporosis drugs	M05BX, H05AA, H05BA	
	Other hormone therapies	H03AA, L02AE,G03XC,L02BG	
	Barbiturates, antipsychotic and antiepileptic drugs	N03AA, N03AB, N03AD, N03AE, N03AF, N03AG,N03AX,N05AA, N05AB,N05AC,N05AD,N05AE, N05AF,N05AG,N05AN,N05AX,	
	Antihistamines	R06AA, R06AB, R06AC, R06AE, R06AE, R06AX	
	Oestrogens	G03CA, G03FA, G03FB	

ATC: Anatomical, Therapeutic, Chemical classification system; ICD-9 or ICD-10: International Statistical Classification of Diseases, version 9 or version 10.

Study participants were divided into two groups (Fig. 2): BIPHOS, consisting of the women who had retrieved a prescription from their pharmacy for bisphosphonates, with or without calcium and vitamin D, and CalVitD, the control group of women who had only taken calcium and vitamin D.

### Data sources

Drug information was obtained from the HRL Pharmacy Unit, which has collected data on all HRL prescriptions dispensed by pharmacies since 2002. Primary care centres managed by the Catalan Institute of Health provide free universal healthcare to 95% of the population of this HRL; during the study period, pharmaceuticals were also provided free of charge to patients older than 65 years and at a 60% subsidy to younger patients.

Baseline information about fractures and co-morbidities was extracted from two sources: the primary care system’s database of electronic health records and the hospital discharge databases of the HRL’s two referral hospitals. The pharmacy database was cross-referenced with the diagnostic records of the HRL’s two hospitals and the primary care centres to obtain baseline and follow-up information.

### Ethical aspects

We carefully respected all the Helsinki Declaration criteria. Since this was an observational study, participants underwent no interventions other than usual clinical care. Information from clinical records was correctly anonymized before analysis in order to preserve the participants’ confidentiality, in accordance with Spanish law (Ley Orgánica 15/1999, Protección de Datos de Carácter Personal). The study protocol was approved by Clinical Ethics Committee of the Primary Healthcare University Research Institute IDIAP-Jordi Gol (P11/85) (S1 Fig).

### Sample, matching process, and statistical power

**Fig 3:**
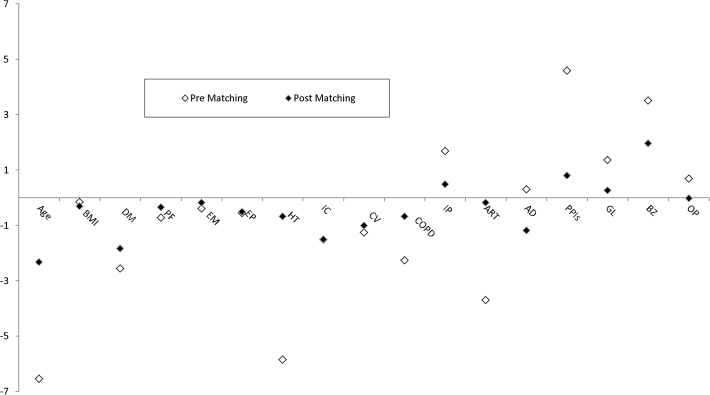
Absolute differences between groups (BIPHOS-CalVitD) pre and post matching. BMI: Body mass index; DM: Type II diabetes mellitus; PF: Behavioural syndromes associated with physiological disturbances and physical factors; EM: Extrapyramidal and movement disorders; EP: Episodic and paroxysmal disorders; HT: Hypertension; IC: Ischaemic heart diseases; CV: Cerebrovascular disease; COPD: Chronic lower respiratory diseases; IP: Inflammatory polyarthropathies; ART: Arthrosis; AD: Antidepressants; PPIs: Proton-pump inhibitors; GC: Glucocorticoids; BZ: Benzodiazepine; OP: Opiates.

The two study groups were matched to ensure balance in terms of basal comorbidities, age, and use of other drugs that modify bone metabolism (Table 2, Fig. 3). Matching was done by the “Nearest Neighbour algorithm” (caliper = 0.1), using the "MatchIt" library of the R (v3.0.1) statistical package [27,28]. The Nearest-Neighbour matching algorithm was employed to find as many matches between groups based on the propensity scores to produce two balanced patient cohorts. The distance was created with the link logit according the following variables: Age, Comorbidity (Hypertension, Chronic lower respiratory diseases, Inflammatory polyarthropathies, Arthrosis), and Drugs prescription (Proton-pump inhibitors and Glucocorticoids).The final matched sample included 1208 women, 604 per group; after the matching process, the potential selection bias between the two samples (total vs. matched) was reduced by 69%. The selection bias reduction was computed according the overall difference between the matched sample and pre-matched sample regarding the sum of relative differences (between exposure groups) in the variables represented in Fig. 3. Assuming a minimum absolute risk reduction of 5% (15% vs. 10% incidence)[14] in a sample of 1208 women, we obtained a statistical power approximated of 91%, with an alpha level of 0.05 and standard deviation of random effect at cluster level of 0.9 (according our data analysis) using logistic regression test with sandwich robust standard (This approximation was performed with R simulation code done by Arnold B.F., 2011 [29].

**Table 2:** Baseline characteristics of the participants according to the group.

Variable		CalVitD (n = 604)		BIPHOS (n = 604)		p value
	Category	n	(%)	n	(%)	
Age group						0.236^a^
	60–64	113	(18.7)	98	(16.2)	
	65–74	230	(38.1)	259	(42.9)	
	>74	261	(43.2)	247	(40.9)	
Age	Mean ± SD	73.5	±8.6	73.1	±7.9	0.384^b^
BMI	Mean ± SD	29.1	±4.3	28.8	±4.1	0.903^b^
Comorbidity						
	Diabetes mellitus	94	(15.6)	83	(13.7)	0.377^a^
	Behavioural syndromes associated with physiological disturbances and physical factors	6	(1.0)	4	(0.7)	0.530^a^
	Extrapyramidal and movement disorders	9	(1.5)	8	(1.3)	0.808^a^
	Episodic and paroxysmal disorders	9	(1.5)	6	(1.0)	0.409^a^
	Hypertensive diseases	277	(45.9)	273	(45.2)	0.814^a^
	Ischemic heart diseases	23	(3.8)	14	(2.3)	0.133^a^
	Cerebrovascular diseases	17	(2.8)	11	(1.8)	0.262^a^
	Chronic lower respiratory diseases	36	(6.0)	32	(5.3)	0.602^a^
	Inflammatory polyarthropathies	16	(2.6)	19	(3.1)	0.579^a^
	Arthrosis	75	(12.4)	74	(12.3)	0.927^a^
Drugs, number of boxes dispensed (mean ± SD)						
	Antidepressants	15.9	±32.5	14.7	±31.3	0.525^c^
	Proton-pump inhibitors	24.4	±31.6	25.3	±31.1	0.655^c^
	Glucocorticoids	3.4	±11.5	3.7	±11.2	0.669^c^
	Benzodiazepine	31.6	±51.5	33.6	±49.1	0.496^c^
	Opiates	6.0	±28.0	6.0	±19.0	0.995^c^

n: Frequency; SD: Standard deviation; P value computed using: Univariate logistic regression with robust standard errors (a); Mixed linear regression by pairs(b) and Mann-Whitney test (c).

### Length of follow-up

Time free of fracture was defined as the time between the first dispensation of medication in 2005 until the first fracture recorded by the primary care doctor, or hospital admission or urgent care visit for fracture, or abandonment. Reasons for abandoning the study (lost to follow-up) were death, change of address, or final medical contact in the HRL’s records before 31 December 2010 (Figs. 1 and 2).

### Primary outcomes

The primary event was defined as first fracture. Osteoporosis-related fracture diagnoses were selected. They were coded according to the International Classification of Diseases (ICD-10 or ICD-9): Fracture of femur; Osteoporosis, pathological fracture, Fatigue fracture of vertebra; Fracture of lower end of radius; and Fracture of upper end of humerus (Table 1).

### Drug exposure

For each patient, the number of boxes of medication dispensed with HRL prescriptions, mainly oral bisphosphonates, calcium, and vitamin D, was calculated from initiation to the last date of follow-up. Drugs were coded according to the Anatomical Therapeutic Chemical Classification System (ATC). Table 1 presents the remaining drug variables as well as the other co-variables analysed.

### Statistical methods

Initially, basal characteristics of both groups were evaluated to establish homogeneity in age, comorbidities, and exposure to other drugs that modify bone metabolism. The incidence of fracture and accumulated risk of fracture after five years was calculated for each group (BIPHOS vs. CalVitD). To evaluate time-related incidence curves, we performed Cox regression models. Risk functions and hazard ratios (HR) with their 95% confidence interval (95%CI) were estimated to compare the BIPHOS group to the CalVitD group. The 95% confidence intervals and p values was computed with robust standard errors to account the matched sample. The models were constructed using co-variables that were clinically adjusted and/or statistically associated with fracture risk. We evaluated goodness-of-fit and the Cox model’s proportional risk assumption, as well as the interactions at different levels of exposure to each drug, using the Schoenfeld residual analysis. A secondary analysis of the sensitivity of the estimated HR for drug exposure levels was carried out, considering the number of boxes of medication collected at the pharmacy: low (≤12), moderate (13–36), and high (>36). The stability and consistency of the models was evaluated using various subsamples of patients whose doctors meet high-quality standards for data entry in the medical records system (22% of the sample). This quality sample (SIDIAP-Q database) minimizes the risk of global bias in epidemiological studies and improves representativeness, as previously published in a validation study[30]. Statistical significance was established as a *p*-value < 0.05. Data management and analysis was done with the SPSS (v17) and STATA v11-IC statistical packages.

## Results

Sociodemographic characteristics of the study participants are shown in Table 2; there were no significant differences between the two study groups. At the beginning of follow-up, the mean age of participants was 73.3 years (SD = 8.3) and body mass index (BMI) was about 29 (SD = 4.2). The most prevalent pathology was hypertension (45%), followed by diabetes (14.7%).

During a mean follow-up of 4.87 years, 138 fractures were recorded, representing an accumulated risk after 5 years of 11.4% (95% confidence interval 9.6 to 13.2%). Half of the fractures were of the femur (50.7%), followed by vertebral fractures (27.5%), unspecified osteoporotic fractures (23.9%), and fractures of the humerus or radius (19.5%).

During their fracture-free period, the CalVitD cohort received a mean 11 (SD = 20) boxes of calcium and vitamin D and the BIPHOS cohort received 11.6 (SD = 16.4) boxes, in addition to a mean 22.6 (SD = 24.4) boxes of bisphosphonates.

Table 3 shows the cumulative incidence of fracture at 5-year follow-up by study group, stratified by level of drug exposure and by the co-variables studied. Accumulated risk was 11.8% among women in the BIPHOS group and 11.1% in the CalVitD group (no significant difference between groups). Among women with moderate drug exposure, the BIPHOS group had significantly higher accumulated fracture than the controls. The remaining co-variables –older age, lower BMI, and a basal hypertension diagnosis–were significantly associated with increased risk of fracture.

**Table 3:** Frequency and fracture risk according to study group and basal comorbidity.

	Risk at 5 years			
Variable	Fractures		CI 95%	
Category	n	(%)	(L inf to L Sup)	p value^a^
Group				
CalVitD: Calcium + Vitamin D	71	(11.8)	(9.2 to 14.3)	0.710
BIPHOS: Bisphosphonate	67	(11.1)	(8.6 to 13.6)	
Exposure level: Number of boxes dispensed				
Low: ≤12				
CalVitD: Calcium + Vitamin D	60	(12.9)	(9.9 to 16.0)	0.604
BIPHOS: bisphosphonate	36	(11.7)	(8.1 to 15.3)	
Moderate: 13–36				
CalVitD: Calcium + Vitamin D	5	(5.4)	(0.8 to 10.1)	0.017
BIPHOS: bisphosphonate	21	(15.9)	(9.7 to 22.1)	
High: >36				
CalVitD: Calcium + Vitamin D	6	(12.5)	(3.1 to 21.9)	0.155
BIPHOS: bisphosphonate	10	(6.1)	(2.4 to 9.8)	
Age group at baseline				
60–64	8	(3.8)	(1.2 to 6.4)	<0.001
65–74	40	(8.2)	(5.8 to 10.6)	
>74	90	(17.7)	(14.4 to 21.0)	
Body mass index at baseline				
≤ 25.00	23	(13.1)	(8.1 to 18.0)	0.482
25.01–30.00	75	(11.9)	(9.4 to 14.4)	
30.01+	39	(9.9)	(6.9 to 12.8)	
Basal comorbidity				
Diabetes mellitus	23	(13.0)	(8.0 to 17.9)	0.465
Behavioural syndromes associated with physiological disturbances and physical factors	5	(50.0)	(19.0 to 81.0)	<0.001
Extrapyramidal and movement disorders	3	(17.6)	(-0.5 to 35.8)	0.417
Episodic and paroxysmal disorders	3	(20.0)	(-0.2 to 40.2)	0.265
Hypertensive diseases	84	(15.3)	(12.3 to 18.3)	<0.001
Ischemic heart diseases	5	(13.5)	(2.5 to 24.5)	0.688
Cerebrovascular diseases	5	(17.9)	(3.7 to 32.0)	0.282
Chronic lower respiratory diseases	9	(13.2)	(5.2 to 21.3)	0.631
Inflammatory polyarthropathies	6	(17.1)	(4.7 to 29.6)	0.297
Arthrosis	12	(8.1)	(3.7 to 12.4)	0.202

CI 95%: Confidence Interval with 95%; n: Fracture frequency

a: p value computed using univariate logistic regression with robust standard errors by clusters (pairs)

Table 4 shows the crude and adjusted Hazard Ratio (HR) for the specific risk of femoral, radial-humeral, and vertebral fractures. None of the models detected significant differences in fracture risk between groups. The BIPHOS group had a slightly –but not significantly–lower global risk of fracture (HR_crude_ = 0.899 / HR_adj_ = 0.934). In the analysis by level of exposure, women with moderate use of bisphosphonates (13–36 units over 1–3 years) had a higher global risk of fracture (HR = 3.0; 95%CI: 1.13 to 7.9). With respect to fracture typology, the BIPHOS group had a lower risk of femoral fracture (HR_adj_ = 0.73; 0.45 to 1.21) and higher risk of vertebral fracture (HR_adj_ = 1.40; 0.82 to 2.42); again, none of these differences achieved significance (p-value > 0.05).

**Table 4:** Crude risk fracture and adjusted by groups and baseline comorbidity.

*Model*			
Variable	Hazard Ratio	(95% CI)	p value^a^
Category^b^			
*Crude*			
Bisphosphonate group (Ref: CalVitD)	0.899	(0.64 to 1.26)	0.532
*Adjusted*			
Bisphosphonate group (Ref:CalVitD)	0.934	(0.67 to 1.30)	0.687
Age (in years)	1.075	(1.05 to 1.10)	<0.001
Body mass index	0.953	(0.91 to 1.00)	0.034
Comorbidity (Ref = No)			
Hypertensive diseases	1.705	(1.20 to 2.42)	0.003
Cerebrovascular diseases	1.323	(0.54 to 3.23)	0.538
Arthrosis	0.641	(0.34 to 1.21)	0.169
Drug therapies at baseline (1 year previous)			
Proton-pump inhibitors	0.962	(0.66 to 1.41)	0.844
Glucocorticoids	1.234	(0.74 to 2.06)	0.422
Antidepressants	1.159	(0.77 to 1.75)	0.482
Benzodiazepine	0.838	(0.58 to 1.20)	0.334
Opiates	1.396	(0.89 to 2.20)	0.149
**Specific fractures**			
***Femur***			
Crude			
Bisphosphonate group (Ref: CalVitD)	0.742	(0.46 to 1.20)	0.227
Adjusted			
Bisphosphonate group (Ref: CalVitD)	0.735	(0.45 to 1.21)	0.228
***Upper humeral or lower radial epiphysis***			
Crude			
Bisphosphonate group (Ref: CalVitD)	0.867	(0.40 to 1.87)	0.716
Adjusted			
Bisphosphonate group (Ref: CalVitD)	0.883	(0.41 to 1.91)	0.752
***Vertebral fracture*: *fatigue or unspecified***			
Crude			
Bisphosphonate group (Ref: CalVitD)	1.256	(0.66 to 2.38)	0.484
Adjusted			
Bisphosphonate group (Ref: CalVitD)	1.405	(0.82 to 2.42)	0.219

a: p values, and Confidence Interval according the Cox regression model with robust standard errors

b: Reference category is CalVitD group, and not presence of comorbidity; 95% CI: Confidence Interval with 95%.

**Fig 4:**
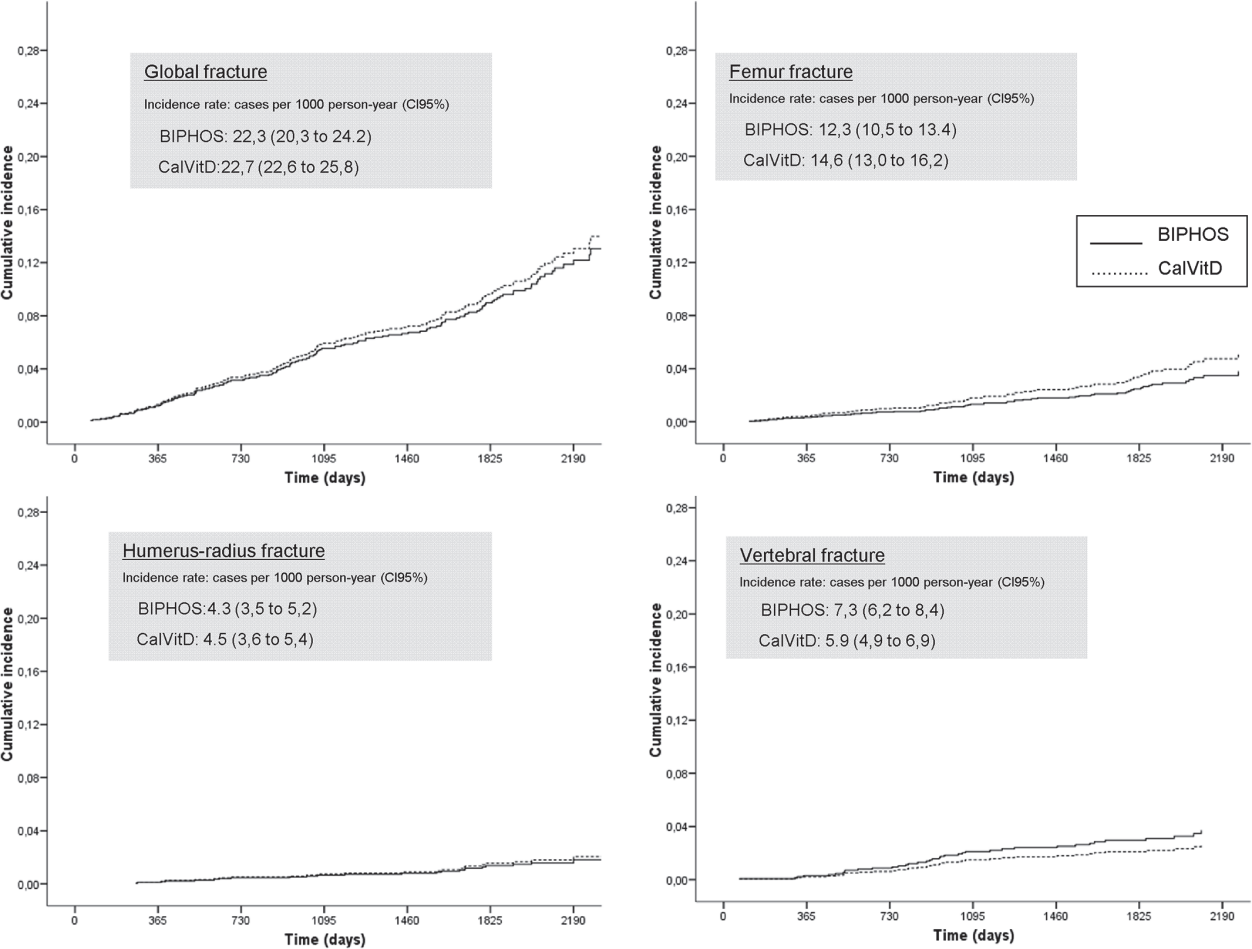
Incidence curves of fracture during 5 years follow-up by group (BIPHOS / CalVitD).

Fig. 4 shows the incidence curves of global and site-specific fractures, adjusted by basal characteristics, for both study groups. In humeral or radial long bones, the curves are practically superimposed; in the femur, the CalVitD curve is slightly higher (but non-significant) and in vertebral fractures the BIPHOS curve is higher.

## Discussion

In this study, which included 1208 women aged 60 years and older without previous fracture who were receiving drug therapy as primary prevention, the cumulative incidence of fracture during five years of follow-up was 11.4% (9.6 to 13.2%). One in 10 participants presented with some type of fracture during the study period, half of them femoral fractures; this corresponds to a 10-year incidence of 22 osteoporosis-related fractures per 100 women.

The two cohorts were selected so that the only distinguishing feature was whether or not they were taking bisphosphonates. They were of comparable age, BMI, comorbidities associated with fracture risk, and use of other prescription drugs. Under these conditions, we did not find any decreased risk of fracture between the groups during the five years of follow-up.

The appearance of a first fracture was associated with advanced age (the main risk factor described in the literature), lower BMI, and a history of hypertension[23]. The first two are well known, particularly in secondary prevention, and are included in the FRAX and QFracture risk equations[9,10]. The hypertension association could be due to the relationship between use of hypertension medications and increased falls, especially at treatment initiation and in elderly patients [31]. We did not find any risk reduction with respect to the site of fractures (femoral, vertebral, humeral neck or head), as shown in Fig. 4.

The FIT-2 study, one of the few clinical trials with a large group of postmenopausal women (n = 4432, mean age, 68 years), compared alendronate with placebo in primary prevention. At four years of follow-up, differences in clinical (vertebral and non-vertebral) and hip fractures did not reach significance. However, a significantly lower incidence of radiological vertebral fractures was observed in the group treated with alendronate [32].

A high percentage of asymptomatic spinal fractures, also called morphometric fractures, occur; these are usually found during exploratory exams and have limited impact on quality of life [31]. Under real-life conditions, asymptomatic fractures are likely under-diagnosed and under-reported in the electronic health records, which would help to explain why no differences were observed in our study.

Given the findings about bisphosphonates treatment in primary prevention, can we justify the investment in preventing only one type of fractures that are asymptomatic and have limited impact on quality of life? We cannot forget the secondary effects of any preventive effort, especially if drug therapy is involved. The use of bisphosphonates has been associated with increased risk of mandibular osteonecrosis, osteoarticular pain, atrial fibrillation, sub-trochanteric and diaphyseal fractures of the femur, and esophagitis [33].

In concordance with the present results, the Catalan Agency for Healthcare Quality and Assessment (*Agencia per la Qualitat i Avaluació Sanitàries Catalana*) advises against the use of bisphosphonates in postmenopausal women with low risk of fracture because the benefits do not exceed the risks[34]. In addition, the 2010 report from Spain’s Agency for the Evaluation of Healthcare Technologies (AETS, *Instituto de Salud Carlos III*) concluded that, assuming partial adherence to treatment, none of the drug interventions evaluated in comparison with calcium plus vitamin D or placebo obtained acceptable cost-utility outcomes if treatment was initiated before 69 years of age [25].

In 2010, the United Kingdom’s Secretary of State issued a report that analysed variations in drug uses in 14 countries. Spain was first on the list in use of osteoporosis medications, even though it had one of the lowest levels of osteoporotic fracture risk [35].

Various scientific societies agree on eradicating low-value clinical practices, such as systematic primary prevention CT scans of postmenopausal women without other risk factors (*Compromiso por la calidad de las Sociedades Científicas*, Ministry of Health, Social Policy and Equality, Spain),[36] in women younger than 65 years without other risk factors (Choosing Wisely, American Academy of Family Physicians) [37], or without evaluating risk using the FRAX or QFracture equations (Do not do, UK National Institute of Health and Care Excellence (NICE) [38].

With the current trend of population aging, the incidence of fractures associated with osteoporosis is expected to increase. Prevention is an objective for all healthcare systems, focused mainly on hip fractures and followed by clinical vertebral fractures because of their implications for morbidity, mortality, and quality of life. In any case, any preventive interventions must be undertaken with caution because, as stated above, they target healthy individuals and large segments of the population [22].

### Strengths and limitations

We are aware that our study has several potential limitations that preclude us from providing definitive evidence of the absence of benefit or the association between bisphosphonates use and the risk of fracture in comparison with calcium and vitamin D. Clinical records from a large population database bring with them implicit biases related to under-reporting. To minimize this limitation, in addition to the fractures included in the primary care electronic records we included those that caused a visit to urgent care or a hospital admission in the region’s two reference hospitals. The availability of this large population database (Fig. 2) allowed us to set very high standards for the final participant selection, eliminating potential selection biases and allowing the inclusion of homogeneous cohorts. We included only current users of the healthcare system (i.e., active records), evidenced by follow-up visits, and without a known history of fracture or previous use of osteoporosis treatment or oestrogens. Finally, strict matching was performed to construct balanced study groups (Table 2 and Fig. 3), which reduced the potential selection bias by 69%.

The total sample used for analysis consisted of 1208 women. This sample size might not be sufficient to detect actual reductions in the incidence of fractures observed in our study (<7% over 5 years: HR = 0.934), but it has sufficient power to detect a reduction of 20% or more in the risk of fracture, as reported in the literature [32].

We know that collecting drugs from a pharmacy with a doctor’s prescription does not provide a precise measurement of the use of the medication, although we assumed that this was evidence of treatment adherence.

An important strength of our study is that the evaluation of the potential effectiveness of bisphosphonates use was done under actual conditions of daily clinical practice, unlike the structure of clinical trials [21]. Furthermore, the availability of a subsample of electronic health records in a database that has been validated for high-quality coding by clinicians reinforces the validity of our results.

## Conclusions

In our study, postmenopausal women obtained no benefit from primary prevention with bisphosphonates in reducing their 5-year risk of first osteoporosis-related fracture, compared to treatment only with calcium plus vitamin D. There was also no risk reduction according to fracture site (femur, vertebrae, or humeral head or neck).

If bisphosphonates use is not shown to have better outcomes than calcium plus vitamin D, primary prevention strategies shall be reconsidered and one should stop doing what is not effective. Medical societies must work together to unify their criteria, reduce the inappropriate use of tests and treatments [18], and use BMD scores appropriately [33]. The availability of an objective measurement simplifies clinical decision-making, which is probably the reason BMD has been used as the measure indicating the prescription of osteoporosis therapy and as a diagnostic method in women younger than 60 years.

In clinical practice, emphasis should be focused to improvement secondary prevention and to identification of those patients at high risk and, therefore, would benefit from a primary prevention therapy. It may be stressed that BMD, per se, is simply a risk factor to be considered, not the identifier by which patients should be selected for treatment.

## Supporting Information

S1 Dataset Data set in Stata format (dta). Data set from 1208 records including: Group, Follow-up time, Fracture, Fracture femur, Fracture of humeral/radial, Fracture vertebral, Propensity distance group, Age, BMI, Hypertensive diseases, Cerebrovascular diseases, Arthrosis, Proton-pump inhibitors, Glucocorticoids, Antidepressants, Benzodiazepine, Opiates, BMI Group, Age group, Diabetes mellitus, Behavioural syndromes associated with physiological disturbances, Extrapyramidal and movement disorders, Episodic and paroxysmal disorders, Ischaemic heart diseases, Chronic lower respiratory diseases, Inflammatory polyarthropathies, Exposure level.
(DTA)

S1 Fig Statement from the clinical investigation ethics. (PDF)

S1 Table STROBE Statement—Checklist of items that should be included in reports of cohort studies. (DOCX)

## References

[1] Pages-Castella A, Prieto Alhambra D. Degenerative osteoarthritis, osteoporosis and fractures: Controversies and evidences. Med Clin (Barc). 2013; 141: 217–220. 10.1016/j.medcli.2013.01.036 23540390

[2] Vazquez M. Osteoporosis: The crisis of a paradigm. Med Clin (Barc). 2010; 134: 206–207. 10.1016/j.medcli.2009.10.009 19939415

[3] WHO Study Group. Assessment of fracture risk and its application to screening for postmenopausal osteoporosis. report of a WHO study group. World Health Organ Tech Rep Ser. 1994; 843: 1–129. 7941614

[4] Pressman A, Forsyth B, Ettinger B, Tosteson AN. Initiation of osteoporosis treatment after bone mineral density testing. Osteoporos Int. 2001; 12: 337–342. 10.1007/s001980170099 11444079

[5] Azagra R, Roca G, Encabo G, Aguye A, Zwart M, et al FRAX(R) tool, the WHO algorithm to predict osteoporotic fractures: The first analysis of its discriminative and predictive ability in the spanish FRIDEX cohort. BMC Musculoskelet Disord. 2012; 13: 204-2474-13-204. 10.1186/1471-2474-13-204 PMC3518201 23088223

[6] Kanis JA, Johnell O, Oden A, Sembo I, Redlund-Johnell I, et al Long-term risk of osteoporotic fracture in malmo. Osteoporos Int. 2000; 11: 669–674. 11095169 10.1007/s001980070064

[7] Sanfelix-Genoves J, Catala-Lopez F, Sanfelix-Gimeno G, Hurtado I, Baixauli C, et al Variability in the recommendations for the clinical management of osteoporosis. Med Clin (Barc). 2014; 142: 15–22. 10.1016/j.medcli.2012.10.025 23332628

[8] Casado E, Caamano M, Sanchez-Burson J, Salas E, Malouf J, et al Management of the patient with a high risk of fracture in clinical practice. results from a survey of 174 spanish rheumatologists (OSTEOPAR project). Reumatol Clin. 2011; 7: 305–313. 10.1016/j.reuma.2010.12.008 21925446

[9] Kanis JA, Johnell O, Oden A, Johansson H, McCloskey E. FRAX and the assessment of fracture probability in men and women from the UK. Osteoporos Int. 2008; 19: 385–397. 10.1007/s00198-007-0543-5 18292978 PMC2267485

[10] Hippisley-Cox J, Coupland C. Predicting risk of osteoporotic fracture in men and women in england and wales: Prospective derivation and validation of QFractureScores. BMJ. 2009; 339: b4229 10.1136/bmj.b4229 19926696 PMC2779855

[11] Gonzalez Lopez-Valcarcel B, Sosa Henriquez M. Estimate of the 10-year risk of osteoporotic fractures in the spanish population. Med Clin (Barc). 2013; 140: 104–109. 10.1016/j.medcli.2011.11.030 22401729

[12] Wells GA, Cranney A, Peterson J, Boucher M, Shea B, et al Etidronate for the primary and secondary prevention of osteoporotic fractures in postmenopausal women. Cochrane Database Syst Rev. 2008; (1):CD003376. doi: CD003376. 10.1002/14651858.CD003376.pub3 18254018 PMC6999803

[13] Wells G, Cranney A, Peterson J, Boucher M, Shea B, et al Risedronate for the primary and secondary prevention of osteoporotic fractures in postmenopausal women. Cochrane Database Syst Rev. 2008; (1):CD004523. doi: CD004523. 10.1002/14651858.CD004523.pub3 18254053

[14] Wells GA, Cranney A, Peterson J, Boucher M, Shea B, et al Alendronate for the primary and secondary prevention of osteoporotic fractures in postmenopausal women. Cochrane Database Syst Rev. 2008; (1):CD001155. doi: CD001155. 10.1002/14651858.CD001155.pub2 18253985

[15] Guerra-Garcia MM, Rodriguez-Fernandez JB, Puga-Sarmiento E, Charle-Crespo MA, Gomes-Carvalho CS, et al Incidence of hip fractures due to osteoporosis in relation to the prescription of drugs for their prevention and treatment in galicia, spain. Aten Primaria. 2011; 43: 82–88. 10.1016/j.aprim.2010.04.010 20554353 PMC7025055

[16] Arias LM, Treceno C, Garcia-Ortega P, Rodríguez-Paredes J, Escudero A, et al Hip fracture rates and bisphosphonate consumption in spain. an ecologic study. Eur J Clin Pharmacol. 2013; 69: 559–564. 10.1007/s00228-012-1337-z 22821192

[17] Álvarez Rodríguez E. Optimización del tratamiento con alendronate en osteoporosis [Improvement of treatment with alendronate in osteoporosis]. Universidad Complutense de Madrid. 2009; Available: http://eprints.ucm.es/8891/1/T30917.pdf. Accessed 04 April 2014

[18] Sanfelix-Gimeno G. Opportunities for improvement in the management of osteoporosis. time to tackle the essential. Med Clin (Barc). 2013; 141: 527–528. 10.1016/j.medcli.2013.09.013 24210981

[19] Imaz I, Zegarra P, Gonzalez-Enriquez J, Rubio B, Alcazar R, et al Poor bisphosphonate adherence for treatment of osteoporosis increases fracture risk: Systematic review and meta-analysis. Osteoporos Int. 2010; 21: 1943–1951. 10.1007/s00198-009-1134-4 19967338

[20] Park-Wyllie LY, Mamdani MM, Juurlink DN, Hawker GA, Gunraj N, et al Bisphosphonate use and the risk of subtrochanteric or femoral shaft fractures in older women. JAMA. 2011; 305: 783–789. 10.1001/jama.2011.190 21343577

[21] Giannini S, Varenna M. Observational studies in osteoporosis treatment. Reumatismo. 2009; 61 Suppl 2: 2–10. 19999185

[22] Migliore A, Broccoli S, Massafra U, Cassol M, Frediani B. Ranking antireabsorptive agents to prevent vertebral fractures in postmenopausal osteoporosis by mixed treatment comparison meta-analysis. Eur Rev Med Pharmacol Sci. 2013; 17: 658–667. 3443 [pii]. 23543450

[23] Casado E. Nuevos datos sobre el tratamiento con bifosfonatos: ¿Son aconsejables unas vacaciones terapeúticas? [New data on bisphosphonate therapy: Is a therapeutic advisable vacation?]. Reumatol Clin. 2011; 7: S28–S33. 10.1016/j.reuma.2011.10.004 21924217

[24] Agirrezabala J, Aizpurua I, Albizuri M, Iciar A, Armendáriz M, et al Osteoporosis postmenopáusica: ¿estamos previniendo las fracturas? [Postmenopausal osteoporosis: Are we preventing fractures?]. Infac. 2006; 14: 43–48.

[25] Imaz Iglesia I, Rubio González B, López Delgado M, Amate Blanco J, Gómez Pajuelo P, et al. Análisis coste-utilidad de los tratamientos farmacológicos para la prevención de fracturas en mujeres con osteoporosis en España. [cost-utility analysis of the prevention drug treatment of fractures in osteoporosis women in Spain]. Madrid: Agencia de Evaluación de Tecnologías Sanitarias—Instituto de Salud Carlos III. 2010; Available: http://gesdoc.isciii.es/gesdoccontroller?action=download&id=14/09/2012-3fdd17b5be. Accessed 04 April 2014.

[26] Bolibar B, Fina Aviles F, Morros R, Garcia-Gil Mdel M, Hermosilla E, et al SIDIAP database: Electronic clinical records in primary care as a source of information for epidemiologic research. Med Clin (Barc). 2012; 138: 617–621. 10.1016/j.medcli.2012.01.020 22444996

[27] Daniel H, Imai K, King G, Stuart E. (2007) Matching as nonparametric preprocessing for reducing model dependence in parametric causal inference. Political Analysis. 2007; 15: 199–236.

[28] Austin PC. Optimal caliper widths for propensity-score matching when estimating differences in means and differences in proportions in observational studies. Pharm Stat. 2011; 10: 150–161. 10.1002/pst.433 20925139 PMC3120982

[29] Arnold BF, Hogan DR, Colford JM Jr, Hubbard AE. Simulation methods to estimate design power: An overview for applied research. BMC Med Res Methodol. 2011; 11: 94-2288-11-94. 10.1186/1471-2288-11-94 21689447 PMC3146952

[30] Garcia-Gil Mdel M, Hermosilla E, Prieto-Alhambra D, Fina F, Rosell M, et al Construction and validation of a scoring system for the selection of high-quality data in a Spanish population primary care database (SIDIAP). Inform Prim Care. 2011; 19: 135–145. 22688222 10.14236/jhi.v19i3.806

[31] Butt DA, Mamdani M, Austin PC, Tu K, Gomes T, et al The risk of falls on initiation of antihypertensive drugs in the elderly. Osteoporos Int. 2013; 24: 2649–2657. 10.1007/s00198-013-2369-7 23612794

[32] Cummings SR, Black DM, Thompson DE, Applegate WB, Barrett-Connor E, et al Effect of alendronate on risk of fracture in women with low bone density but without vertebral fractures: Results from the fracture intervention trial. JAMA. 1998; 280: 2077–2082. joc80627 [pii]. 9875874 10.1001/jama.280.24.2077

[33] Jamart Sáncheza L, Herrero Hernándeza S, Barreda Velázqueza C. ¿Está justificado el gasto en fármacos contra la osteoporosis? [Is it justified spending on drugs for osteoporosis?]. MC Form Med Contin Aten Prim. 2011; 18: 317.

[34] Departament de Salut de la Generalitat de Catalunya. Bifosfonats en dones postmenopàusiques amb risc baix de fractures [Bisphosphonates in postmenopausal women with low risk fracture]. 2013; Available: http://www20.gencat.cat. Accessed Abril 2014.

[35] Richards M. A report for the secretary of state for health by professor sir mike richards CBE. COI Crown. 2010; Available: https://www.gov.uk/government/uploads/system/uploads/attachment_data/file/216249/dh_117977.pdf. Accessed 04 April 2014.

[36] Brotons Muntó F, Cerecedo Pérez MJ, González González A, Lázaro Gómez MJ, León Vázquez F, et al. Grupo de trabajo de la semFYC para el proyecto recomendaciones «No hacer» [Working group on recommendations semFYC project "not do"]. e-documentos semFYC. 2014; Available: http://e-documentossemfyc.es/recomendacion-para-no-hacer-de-la-sociedad-espanola-de-medicina-de-familia-y-comunitaria/. Accessed 24 Abril 2014.

[37] American Academy of Family Physicians. Fifteen things physicians and patiens should question. ABIM Foundation. 2014; Available: http://www.choosingwisely.org/doctor-patient-lists/american-academy-of-family-physicians/. Accessed 04 February 2014.

[38] Barry P, Aspray T, Briers K, Collins G, Compston J, et al. 'Do not do' recommendations: Osteoporosis: Assessing the risk of fragility fracture: NICE guideline. 2014; Available: http://www.nice.org.uk/usingguidance/donotdorecommendations/detail.jsp?action=details&dndid=1112. Accessed 20 June 2014.

