# Supplementary material for: Use of Oral Bisphosphonates in Primary Prevention of Fractures in Postmenopausal Women: A Population-Based Cohort Study
Source: PLoS One. 2015 Apr 10;10(4):e0118178. doi: 10.1371/journal.pone.0118178 (PMC4393307; doi:10.1371/journal.pone.0118178)
Supplement: S1 Fig — (PDF) [file pone.0118178.s002.pdf]

STATEMENT FROM THE CLINICAL INVESTIGATION ETHICS

Rosa Morros Pedrós, President of the Clinical Ethics Committee of the IDIAP Jordi Gol and Gurina.

To Certify:

That this Committee in its convocation on September 28th, 2011 evaluated the research project **(P11/85)** titled: ***Incidence of risk of osteoporotic fracture and its relation with bisphosphonates exposure. Observational study of population clinical data: OSTEOFRAC*** presented by Gisela Galindo.

In consideration of the ethical principles and methodology of the investigation, definitive approval has been granted for the abovementioned project.

Signed in Barcelona June 2014.
